# Effectiveness of urine fibronectin as a non-invasive diagnostic biomarker in bladder cancer patients: a systematic review and meta-analysis

**DOI:** 10.1186/s12957-018-1358-x

**Published:** 2018-03-21

**Authors:** Fan Dong, Yifan Shen, Tianyuan Xu, Xianjin Wang, Fengbin Gao, Shan Zhong, Shanwen Chen, Zhoujun Shen

**Affiliations:** 1 Department of Urology, Huashan Hospital, Fudan University, No.12, Middle Urumqi Road, Shanghai, 200040 China; 2 Fudan Institute of Urology, Huashan Hospital, Fudan University, Shanghai, China; 3 Department of Urology, Ruijin Hospital, School of Medicine, Shanghai Jiaotong University, Shanghai, China

**Keywords:** Urine fibronectin, Bladder cancer, Diagnosis, Biomarker, Meta-analysis

## Abstract

**Background:**

Previous researches pointed out that the measurement of urine fibronectin (Fn) could be a potential diagnostic test for bladder cancer (BCa). We conducted this meta-analysis to fully assess the diagnostic value of urine Fn for BCa detection.

**Methods:**

A systematic literature search in PubMed, ISI Web of Science, EMBASE, Cochrane library, and CBM was carried out to identify eligible studies evaluating the urine Fn in diagnosing BCa. Pooled sensitivity, specificity, and diagnostic odds ratio (DOR) with their 95% confidence intervals (CIs) were calculated, and summary receiver operating characteristic (SROC) curves were established. We applied the STATA 13.0, Meta-Disc 1.4, and RevMan 5.3 software to the meta-analysis.

**Results:**

Eight separate studies with 744 bladder cancer patients were enrolled in this meta-analysis. The pooled sensitivity, specificity, and DOR were 0.80 (95%CI = 0.77–0.83), 0.79 (95%CI = 0.73–0.84), and 15.18 (95%CI = 10.07–22.87), respectively, and the area under the curve (AUC) of SROC was 0.83 (95%CI = 0.79–0.86). The diagnostic power of a combined method (urine Fn combined with urine cytology) was also evaluated, and its sensitivity and AUC were significantly higher (0.86 (95%CI = 0.82–0.90) and 0.89 (95%CI = 0.86–0.92), respectively). Meta-regression along with subgroup analysis based on various covariates revealed the potential sources of the heterogeneity and the detailed diagnostic value of each subgroup. Sensitivity analysis supported that the result was robust. No threshold effect and publication bias were found in this meta-analysis.

**Conclusions:**

Urine Fn may become a promising non-invasive biomarker for bladder cancer with a relatively satisfactory diagnostic power. And the combination of urine Fn with cytology could be an alternative option for detecting BCa in clinical practice. The potential value of urine Fn still needs to be validated in large, multi-center, and prospective studies.

**Electronic supplementary material:**

The online version of this article (10.1186/s12957-018-1358-x) contains supplementary material, which is available to authorized users.

## Background

Urinary bladder cancer (BCa), which ranks first in the list of the most life-threatening urinary malignancies, has become a worldwide issue of public health [1]. According to the latest report, almost 20 thousand people die of bladder cancer every year in the USA, not to mention over 79,000 new cases are diagnosed [2]. Among the BCa patients, over 70% suffer from non-muscle-invasive bladder cancer (NMIBC), which can be treated by transurethral resection of bladder tumors (TURBT) and have a much better prognosis than muscle-invasive bladder cancer (MIBC) [3]. However, the tumor can quickly invade the muscle layer and progress frequently to a lethal condition with limited treatment options [4]. Hence, the early diagnosis and intervention of bladder cancer is the key of treating the disease and improving outcomes.

Despite the great progress made in molecular and genetic diagnostics, the screening of bladder cancer is still trapped by the weakness of the current diagnostic methods such as cystoscopy and urine cytology. Cystoscopy is still the standard way for the detection and diagnosis of bladder cancer. But this invasive examining method is costly and may lead to trauma and infection of the urinary system [5]. Urine cytology (Cyto), although non-invasive and specific, has a rather low sensitivity (approximately 35%) with an increased risk of missed diagnosis [6, 7]. To get rid of this predicament, scientists have spent over 20 years looking for a sensitive, specific, and non-invasive biomarker for the detection of bladder cancer. A variety of urinary markers, due to their non-invasiveness and simplicity, have been developed currently [8]. Among them, U.S. Food and Drug Administration has approved bladder tumor antigen (BTA), fibrin/fibrinogen degradation product (FDP), and nuclear matrix protein 22 (NMP 22) for clinically detecting patients with bladder cancer [9].

Fibronectin (Fn) is a large dimeric structural glycoprotein which basically has two biological types, termed plasma and cellular fibronectin [10]. Plasma Fn is synthesized by hepatocytes and released to the circulation while the cellular fibronectin participates in constituting the extracellular matrix and can be found in most of the tissues [11]. In the urinary tract, when tumor exists, fibronectin can be present due to the degradation of the extracellular matrix caused by proteases as well as the leakage from the blood [12–14]. Therefore, the urine Fn may become a potential biomarker for detecting bladder cancer. In 1993, Shen et al. for the first time revealed that the level of urine Fn in bladder cancer patients was significantly higher than that in patients with benign urothelial diseases and the health groups and urine Fn could be utilized as a valuable biomarker for diagnosing bladder cancer [15]. This finding was quickly confirmed in the same year by Malmstrom, who further proved the follow-up value of urine Fn in bladder cancer patients [16]. Since then, the diagnostic value of this urine molecule in detecting bladder cancer has been discussed by various researches and some satisfactory results were reported [17–24]. Recent study showed that urine Fn has a sensitivity of 91.4% and a specificity of 87.8% in detecting residual bladder tumor after TURBT [21]. Moreover, a significantly higher level of urine Fn was found in MIBC patients by some investigators [19, 20]. Although the extensive analyses have been carried out, due to the limited clinical trials, different types of patients, insufficient study populations, and heterogeneous cut-off values, the application of urine Fn in the diagnosis of bladder cancer still needs to be verified, and a detailed evaluation of its diagnostic value would be an essential step before the biomarker’s popularization.

In order to fully analyze the diagnostic performance of urine Fn in bladder cancer patients, we conduct a systematic review with meta-analysis based on eight original researches, which will allow us to sum the relevant researches up and provide more precise estimates of the diagnostic value of urine Fn. Moreover, we also examined whether the combination of urine Fn and urine cytology (Fn+Cyto) can remedy the rather low sensitivity of urine cytology and enhance its diagnostic performance in bladder cancer.

## Methods

### Data sources and search strategy

The systemic review and meta-analysis in this manuscript were performed in accordance with the recommendations of the Quality of Reporting of Meta-analysis (QUOROM) consensus guidelines [25]. A computerized literature search was carried out in PubMed, ISI Web of Science, EMBASE, Cochrane library, and China Biology Medicine disc (CBM) on March 2, 2017, with the following key words and/or medical subject headings: “urine fibronectin” or “fibronectin” or “Fn” plus “bladder cancer” or “bladder tumour” or “urinary bladder neoplasms” or “transitional cell carcinoma,” without language restriction. Besides, references of relevant reviews and retrieved articles were checked for additional articles that were omitted through the database searches. Two investigators (XT and YS) did the searches independently.

### Selection criteria

We search the full-text articles that investigated the effectiveness of urine fibronectin for detection of human beings with bladder cancer. In our meta-analysis, we included researches that met the following criteria: (1) studies utilized urine Fn as a diagnostic test for human bladder cancer patients, (2) the bladder tumor was confirmed by pathology, (3) the urine samples were collected before the final treatments, (5) studies reported the sensitivity (Sen) and specificity (Spe) of urine Fn with their 95% confidence intervals (CIs), gave the number of true positive (TP)/false positive (FP)/false negative(FN)/true negative(TN), or provided sufficient information to calculate them, (6) studies gave clear cut-off value or cut-off criteria. The exclusion criteria were as follows: (1) insufficient data for analysis; (2) case reports, conference reports, retrospective design, reviews, letters, or editorials; (3) duplicate data published in other studies; and (4) full text unavailable.

### Data extraction and quality assessment

Two investigators independently extracted the following data from each article: name of the first author, publication year, study design, study location, total sample size, mean age, number of bladder cancer cases enrolled, number of NMIBC cases, detection method of urine Fn, cut-off criteria, sensitivity, specificity, and area under curve (AUC) of the receiver operating characteristic curve (ROC). The variables including TP, FP, FN, and TN results were also collected or calculated from the data in each article. If there was any disagreement between the two investigators, a discussion among all of the authors would be carried out to resolve it. Quality assessment of the selected studies was conducted using the quality assessment of diagnostic accuracy studies-2 (QUADAS-2), a revised quality assessment tool for systematic reviews of diagnostic studies to evaluate bias in the study [26].

### Statistical analysis

The data analysis was performed by STATA version 13.1 (Stata Corporation, College Station, TX, USA) with the midas and metandi modules as well as Meta-Disc 1.4 (XI Cochrane Colloquium, Barcelona, Spain), which were widely used for meta-analysis of diagnostic studies [27, 28]. RevMan 5.3 (Cochrane Collaboration, Oxford, UK) was used to do the QUADAS-2 assessment. The Sen, Spe, positive likelihood ratio (PLR), negative likelihood ratio (NLR), diagnostic odds ratio (DOR), and diagnostic score, with corresponding 95% CIs, were pooled in order to estimate the diagnostic power of urine Fn in the diagnosis of BCa. We also calculated the summary receiver operating characteristic (SROC) curves and area under the curve (AUC) of urine Fn with 95% confidence contour. To better judge the test performance, hierarchical summary receiver operator curve (HSROC) was constructed by using the metandi module in STATA, which is a flexible method for meta-analysis of diagnostic test accuracy evaluations [29] and is widely used in high-quality researches [30]. Besides, *Z* test was used to judge the diagnostic performance of urine Fn and the combined method (Fn+Cyto). Heterogeneity in the meta-analysis was appraised using Cochran *Q* test (with *p* value) and the *I*^2^ index, with statistically significant heterogeneity set at *P* < 0.05 and *I*^2^ > 50% [31]. To further analyze the sources of heterogeneity, Spearman correlation coefficient test was conducted to rule out the heterogeneity caused by the threshold effect. Moreover, sensitivity analysis, univariable meta-regression, and subgroup analyses were performed. To make better clinical decisions, we calculated post-test probability of bladder cancer through combining the pre-test probability with the likelihood ratios using the Fagan’s nomogram. Deeks’ funnel was used to investigate the publication bias, and plot *P* < 0.05 indicated significant asymmetry [32]. Begg’s rank correlation test was performed as a supplement to estimate the publication bias.

## Results

### Eligible studies

**Fig. 1:**
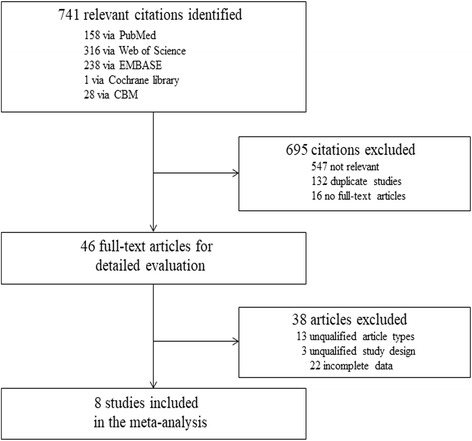
Flow chart of the inclusion and exclusion of the relevant studies

The primary search identified 741 articles from all databases. After excluding the duplicate publications, non-relevant literatures, and articles that did not meet the inclusion criteria, eight manuscripts published between 2000 and 2013 were considered eligible for meta-analysis (Fig. 1) [17–24]. Among the studies, five of them reported the diagnostic value of both urine Fn itself and urine Fn combined with urine cytology (Fn+Cyto) [18, 20, 22–24]. So, we additionally selected those five studies to further evaluate the diagnostic performance of the combination of these two methods (Fn+Cyto).

### Study characteristics

Characteristics of the included studies are shown in Table 1. All studies were carried out from 2000 to 2013 and varied in sample sizes (from 122 to 355), and a total of 744 bladder cancer patients pathologically confirmed at biopsy were included. Of these studies, two were conducted in Asia, two in Europe, and four in transcontinental regions. The pathological types of bladder tumor in three studies [18, 22, 23] consisted of both bladder transitional cell carcinoma (BTCC) and squamous cell carcinoma (SCC) and the rest only included BTCC. In all, three studies gave the volumetric urine Fn concentrations (measurement unit: μg/L), three only reported the Fn cut-offs adjusted by urine creatinine (Cr) (measurement unit: ng/mgCr or ng/μmolCr) in order to eliminate the bias caused by concentrated or attenuated urine samples, and Eissa [23] chose another calibrator—bovine serum albumin (BSA) rather than frequently used urine Cr in one of his studies (ng/mgBSA). Different assay methods were used while the enzyme-linked immunosorbent assay (ELISA) was the most frequently selected. The ROC curves together with respective AUC of urine Fn were reported in six studies. Moreover, we also listed the outcome variables including TP, FP, FN, and TN in Table 1.

**Table 1:** Characteristics of eligible studies

Year	First author	Sample size	Mean age (year)	Patient with BCa	Non-invasive	Assay method	Cut-off criteria	TP	FP	FN	TN	Sensitivity	Specificity	AUC-ROC
2000	Sanchez-Carbayo M	355	NR	130	62	Solid-phase chemiluminescent immunometric assay	52.8 μg/L	104	57	26	168	0.800	0.747	0.823
2002	Eissa S	215	56.5	100	29	ELISA	198 ng/mgCr	83	20	17	95	0.830	0.826	0.836
2003	Mutlu N	130	NR	75	51	Solid-phase chemiluminescent immunometric assay	43.4 ng/mgCr	54	10	21	45	0.720	0.821	0.804
2005	Menendez V	123	NR	68	52	DPC immulite autoanalyzer	25.6 μg/L	53	11	15	44	0.78	0.80	NR
2008	Li LY	167	62.5	126	112	Solid-phase chemiluminescent immunometric assay	67.8 μg/L	49	8	5	60	0.914	0.878	0.896
2010	Eissa S	240	57.76	100	NR	ELISA	186.5 ng/mgCr	82	22	18	118	0.820	0.843	0.920
2011	Eissa S	240	56.60	132	NR	ELISA	41.7 ng/mg BSA	106	42	26	66	0.803	0.612	0.806
2013	Shen ZJ	147	NR	85	64	Gold immunochromatography	Test line of Fn test paper colored	62	13	23	49	0.729	0.790	NR

Note: *NR* not reported, *Cr* urine creatinine, *BSA* bovine serum albumin

### Quality assessment

We used the QUADAS-2 tool to do the study quality assessment, and the results were shown in Additional file 1: Figure S1. The bar graph (Additional file 1: Figure S1A) indicated that the overall quality of the included studies was moderately high. The potential risk of bias mainly came from the patient selections of some studies. To be more precise, the non-random or inconsecutive inclusion of patients might slightly reduce the study quality of this meta-analysis. Additional file 1: Figure S1B listed the performance of each research in the assessment.

### Overall diagnostic accuracy

**Fig. 2:**
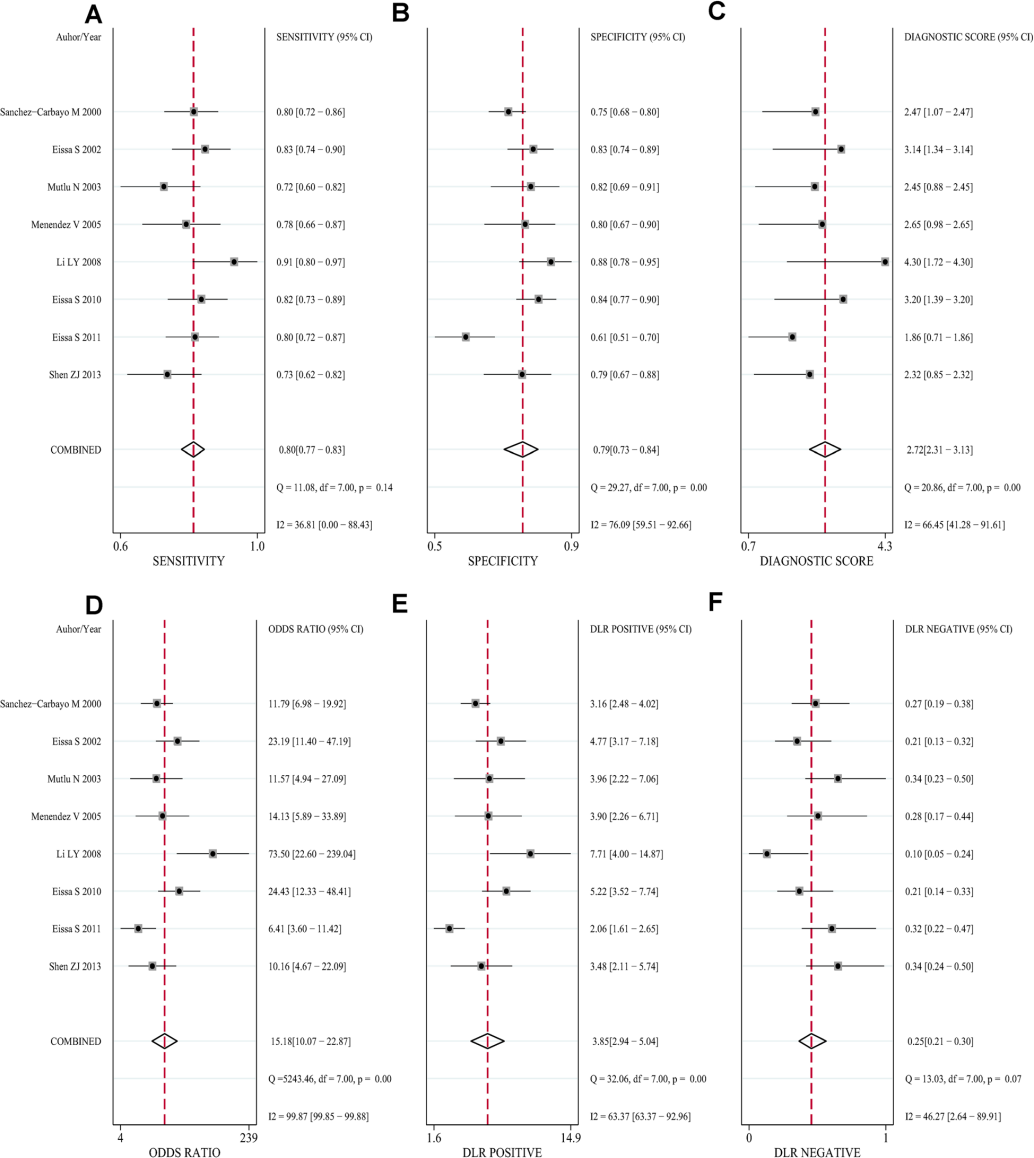
Forest plots of pooled **a** sensitivity, **b** pooled specificity, **c** diagnostic score, **d** odds ratio, **e** positive likelihood ratio, and **f** negative likelihood ratio

**Fig. 3:**
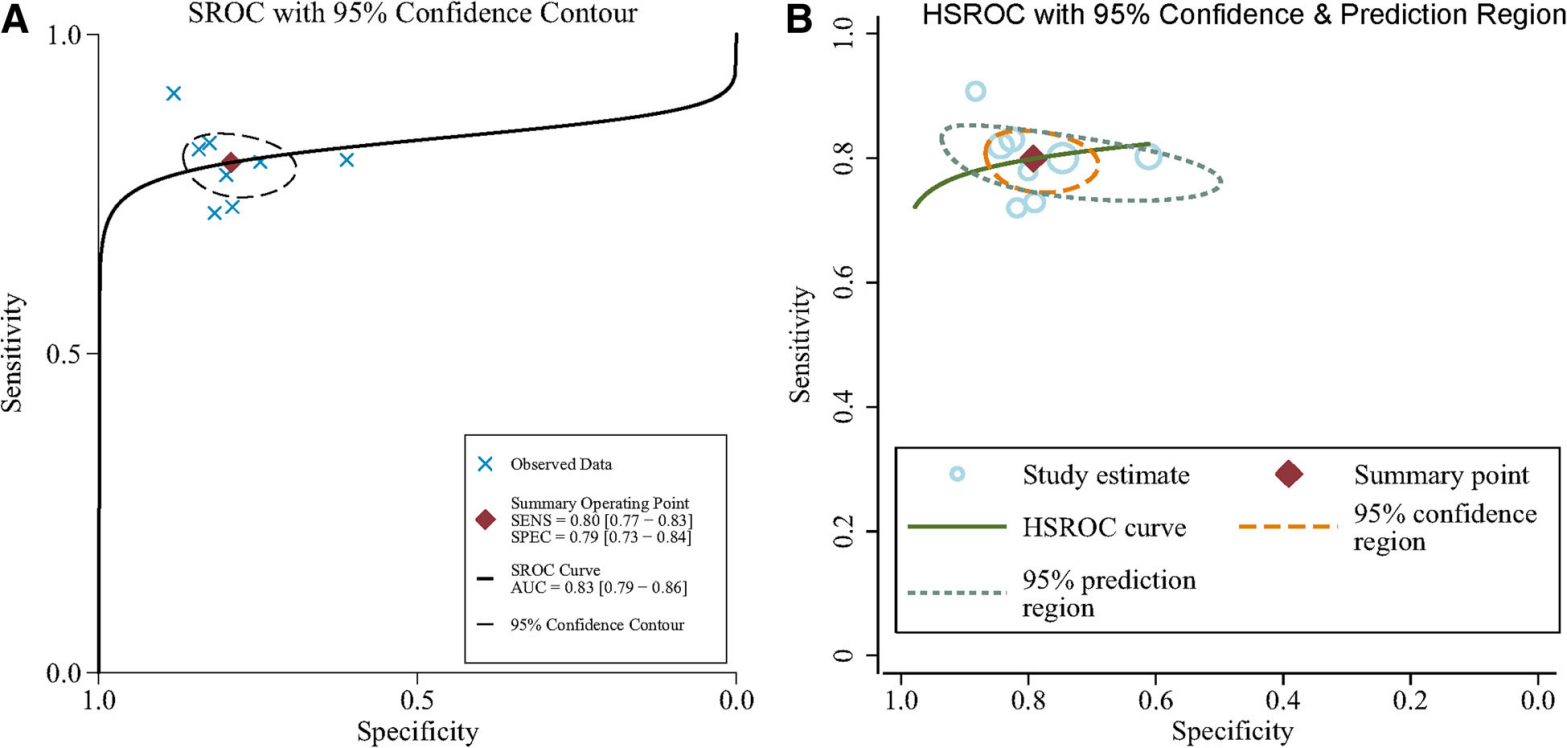
SROC curve and HSROC curve of urine Fn for BCa diagnosis. SROC, summary receiver operating characteristic; HSROC, hierarchical summary receiver operator curves; AUC, area under the curve

**Fig. 4:**
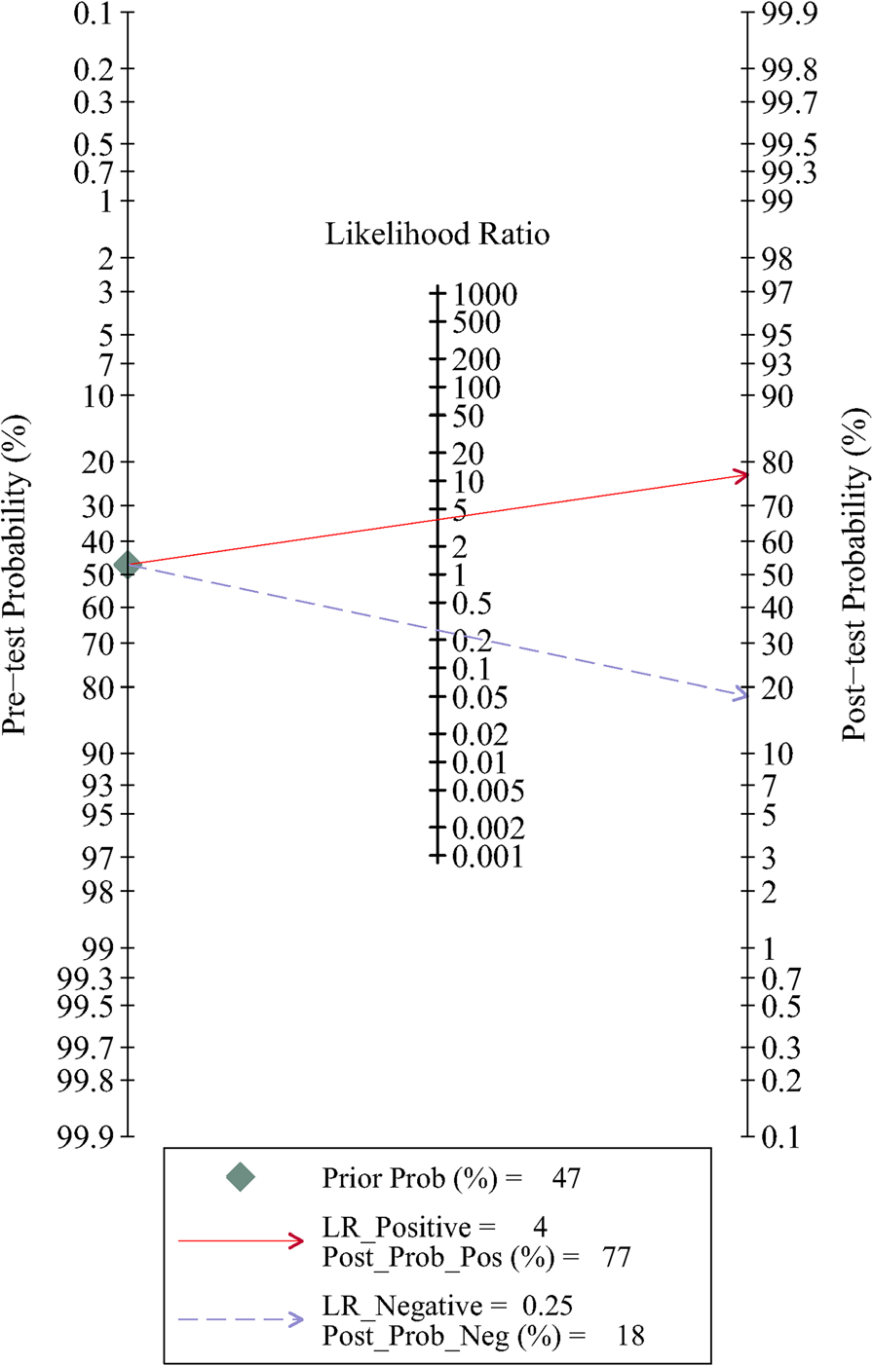
Fagan’s nomogram and post-test probability of urine Fn for the detection of BCa

In our meta-analysis, based on the included studies, the overall pooled sensitivity was 0.80 (95%CI = 0.77–0.83) and the pooled specificity was 0.79 (95%CI = 0.73–0.84), with a DOR of 15.18 (95%CI = 10.07–22.87) and a diagnostic score of 2.72 (95%CI = 2.31–3.13), shown in Fig. 2. The sensitivity and specificity of urine Fn were both high, indicating that this biomarker could not only correctly detect the patients with bladder cancer but effectively avoid the FP cases. The pooled PLR was 3.85 (95%CI = 2.94–5.04), and the NLR was 0.25 (95%CI = 0.21–0.30), shown in Fig. 2e, f, respectively. We also showed the SROC (midas module in STATA) and HSROC (metandi module in STATA) results in Fig. 3a, b. The AUC of SROC was 0.83 (95%CI = 0.79–0.86), which was far greater than 0.70—a reference value of the useful risk predictor in diagnostic tests [33]. The Q* index of this SROC, which referred to the Sen of the point where its Sen was equal to Spe, was 0.798, which further verified the diagnostic accuracy. From both the SROC and HSROC, we could tell that the 95% confidence region of the summary operating point were quite narrow, which illustrated that the confidence level of this summary operating point as a sample to estimate the overall population was sufficiently high. The HSROC also showed a rather restricted 95% prediction region, which was the confidence limit for an individual predicted value of the summary point of urine Fn Sen and Spe. In this study, the pooled pre-test probability was 0.47. Fagan’s nomogram (Fig. 4) of this meta-analysis showed that for patients who were under suspicion for bladder cancer, the probability of developing this disease increased to 77% when urine Fn testing was positive and reduced to 18% when urine Fn testing was negative.

There was no significant heterogeneity between studies as evidenced by a *Q* test *p* = 0.061 and an *I*^2^ index = 54%. Specifically, there was nearly no heterogeneity in Sen (*Q* test *p* = 0.14, *I*^2^ = 36.81%) while an obvious heterogeneity was observed in Spe (*Q* test *p* < 0.05, *I*^2^ = 66.45%). Spearman correlation coefficient of these eight articles was − 0.542 (*p* = 0.183), suggesting there was no significant threshold effect in this meta-analysis. In order to analyze the sources of the heterogeneity in detail and have a better view of the impact of various study characteristics on the diagnostic efficacy of urine Fn, meta-regression along with subgroup analysis were conducted.

### Meta-regression, subgroup analysis, and sensitivity analysis

We considered the (1) urine Fn units of measurement (μg/L or not), (2) the pathological types of bladder cancer (BTCC only or BTCC and SCC), (3) the study objects (primary tumor or residual tumor), (4) the assay methods (ELISA or not), and (5) the proportion of NMIBC in all bladder cancer cases (recorded as > 50% or no evidence) as the potential sources of heterogeneity. First, we used the above-mentioned five covariates to do an univariable meta-regression, and the results were shown in Additional file 2: Figure S2. Except for the covariate “study objects,” the *p* values of other four covariates were all < 0.05 in both Sen and Spe, indicating that those four covariates were the main sources of the heterogeneity. The pooled Sen, Spe, DOR, PLR, NLR, and AUC with their 95% CIs for each subgroup were listed in Table 2 and the respective *I*^2^ were also calculated.

**Table 2:** Subgroup analysis based on various covariates

Subgroup analysis	Category(*n*)	Sen(95%CI)	Spe(95%CI)	DOR(95%CI)	PLR(95%CI)	NLR(95%CI)	AUC-ROC
All		0.80(0.77–0.83)	0.79(0.73–0.84)	15.18(10.07–22.87)	3.85(2.94–5.04)	0.25(0.21–0.30)	0.83(0.79–0.86
	*I*^2^(%)	36.8%	76.1%	99.9%	63.4%	46.3%	
Measurement units	μg/L(3)	0.82(0.76–0.86)	0.78(0.73–0.82)	20.46(7.91–52.90)	4.26(2.58–7.03)	0.23(0.14–0.35)	0.88 (0.80–0.94)
	*I*^2^(%)	53.8%	68.3%	74.3%	70.9%	56.8%	
	Non-μg/L(5)	0.79(0.75–0.82)	0.79(0.70–0.85)	13.46(8.35–21.69)	3.66(2.60–5.16)	0.27(0.22–0.33)	0.80 (0.76–0.83)
	*I*^2^(%)	30.8%	82.4%	98.8%	68.6%	38.51%	
Pathological types	BTCC(5)	0.79(0.73–0.84)	0.80(0.73–0.85)	14.62(8.41–25.41)	3.87(2.85–5.23)	0.26(0.20–0.36)	0.86(0.83–0.89)
	*I*^2^(%)	53.8%	39.4%	99.7%	0.0%	51.5%	
	BTCC&SCC(3)	0.82(0.77–0.86)	0.77(0.72–0.81)	15.09(6.09–37.42)	3.66(1.88–7.14)	0.25(0.18–0.33)	0.89(0.86–0.92)
	*I*^2^(%)	0.0%	90.2%	82.6%	91.0%	35.4%	
Study objects	Primary tumor(7)	0.79(0.76–0.82)	0.78(0.72–0.83)	13.18(9.19–18.91)	3.58(2.78–4.61)	0.27(0.23–0.32)	0.80(0.77–0.84)
	*I*^2^(%)	0.0%	74.4%	97.8%	55.2%	7.3%	
	Residual tumor(1)	0.91(0.71–0.98)	0.88(0.70–0.93)	73.50(22.60–239.04)	0.73(0.55–0.89)	0.93(0.72–0.98)	0.90(0.83–0.92)
Assay methods	ELISA(3)	0.82(0.77–0.86)	0.77(0.72–0.81)	15.09(6.09–37.42)	3.66(1.88–7.14)	0.25(0.18–0.33)	0.89(0.86–0.92)
	*I*^2^(%)	0.0%	90.2%	82.6%	91.0%	35.4%	
	Non-ELISA(5)	0.79(0.73–0.84)	0.80(0.73–0.85)	14.62(8.41–25.41)	3.87(2.85–5.23)	0.26(0.20–0.36)	0.86(0.83–0.89)
	*I*^2^(%)	53.8%	39.4%	99.7%	0.0%	51.5%	
NMIBC > 50%	Yes(4)	0.79(0.70–0.85)	0.83(0.76–0.87)	17.32(8.51–35.28)	4.50(3.11–6.52)	0.26(0.17–0.39)	0.87(0.84–0.90)
	*I*^2^(%)	62.6%	0.0%	99.2%	0.0%	63.8%	
	No evidence(4)	0.81(0.77–0.85)	0.77(0.67–0.84)	14.23(8.02–25.23)	3.48(2.39–5.05)	0.24(0.19–0.31)	0.83(0.80–0.86)
	*I*^2^(%)	0.00%	86.09%	99.9%	75.8%	16.89%	

Note: *BTCC* bladder transitional cell carcinoma, *SCC* squamous cell carcinoma, *NMIBC* non-muscle-invasive bladder cancer, *CI* confidence interval

For measurement units, studies using urine Fn concentration (μg/L) significantly reduced the *I*^2^ index of Spe to less than 70%, without obviously increasing the heterogeneity of Sen. And the diagnostic efficacy of urine Fn using its concentration as the measurement unit was significantly higher than that using other measurement units, with an AUC of 0.88 (95%CI = 0.80–0.94) versus 0.80 (95%CI = 0.76–0.83). As for pathological types, studies based on both BTCC and SCC have a higher Sen with a lower Spe than the studies which only included BTCC cases. Moreover, the “BTCC&SCC” subgroup had an improved AUC [0.89 (95%CI = 0.86–0.92)] than the overall pooled AUC [0.86 (95%CI = 0.83–0.89)], indicating that the urine Fn can be used as a biomarker for not only BTCC. It might also be efficient for detecting SCC. Besides, the AUC of “NMIBC > 50%” were larger than 0.85, which means the Fn might have a very high diagnostic accuracy especially in NMIBC. To evaluate the credibility and consistency of the results, we performed sensitivity analysis by omitting included studies one by one. With sequential removal of each single study, the overall results were essentially unchanged, which further confirmed the robustness of our outcomes.

### Diagnostic performance of urine Fn combined with urine cytology

Among the 11 eligible studies, five also reported the diagnostic value of urine Fn combined with urine cytology (Fn+Cyto) [18, 20, 22–24]. So, we also collected the pooled meta-analysis of bladder cancer patients with the combined testing methods (Fn+Cyto), and the results were summarized in Table 3. In order to better compare the diagnostic efficacy of urine Fn alone and the combined method, we further conducted *Z* tests of these two testing methods and the Z values together with respective *p* values were also listed in Table 3. Among those parameters, the combined method (Fn+Cyto) was significantly more sensitive than urine Fn alone, with a sensitivity of 0.86 (95%CI = 0.82–0.90) versus 0.79 (95%CI = 0.77–0.82) (*p* < 0.01). Moreover, the AUC of the combined method [0.89, 95%CI = (0.86–0.92)] was also significantly larger than that of urine Fn alone [0.82, 95%CI = (0.78–0.85)] (*p* < 0.01), indicating that the combination of Fn and cytology had a much better diagnostic performance than urine Fn alone. However, the Spe and NLR of urine Fn were higher than those of the combined method (*p* < 0.05).

**Table 3:** Meta-analysis of the diagnostic power of the combined method (combination of urine Fn with cytology)

	Test of association		Test of heterogeneity			*Z* test for Fn and combined method	
Parameter	Estimates	95%CIs	*Q*	*p* value	*I*^2^(%)	*Z*	*p* value
Sensitivity	0.86	0.82–0.90	11.48	0.02	65.15%	3.07	0.002
Specificity	0.77	0.70–0.84	17.88	0.00	77.63%	− 2.16	0.031
DOR	21.20	14.30–31.44	20.03	0.00	80.03%	1.52	0.129
Diagnostic score	3.05	2.66–3.45	5.57	0.23	28.24%	1.32	0.187
PLR	3.82	2.87–5.08	16.61	0.00	54.36%	− 0.10	0.920
NLR	0.18	0.13–0.24	8.72	0.07	54.11%	− 2.45	0.014
AUC-ROC	0.89	0.86–0.92				2.75	0.006

### Publication bias

**Fig. 5:**
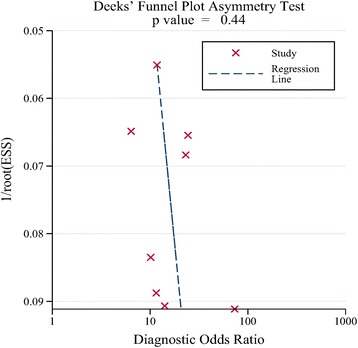
Deeks’ funnel plot with regression line

Deeks’ funnel plot asymmetry test was used to do the publication bias analysis, which showed a symmetric funnel plot in Fig. 5, meaning that there was no significant publication bias among the included studies (Deeks’ test *p* = 0.44). Besides, the Begg’s test was performed and the *z* was 1.36 and *p* value was 0.174, which also proved that no evidence of publication bias for this meta-analysis was observed.

## Discussion

Bladder cancer is a frequent and life-threatening tumor with huge metastasis rate and mortality [34]. However, a non-invasive, timely and accurate diagnosis of BCa remains lacking in our clinical practice. Cystoscopy along with biopsy is still the gold standard for diagnosing BCa, but this invasive testing method has various complications and the results to some extent depend on the samples’ quality and urologists’ as well as pathologists’ experiences. Urine cytology, as a useful adjunct for cystoscopy, often suffers from quite low sensitivity and has atypical results [35, 36]. In order to find a non-invasive and accurate way of detecting BCa and improve the low sensitivity of cytology, many voided urine molecules such as NMP22, BTA, and urine Fn have been developed as non-invasive diagnostic biomarkers for this malignancy. Among those biomarkers, the urine Fn test performance has been well studied since its introduction by Shen and Malmstrom in 1993 [15, 16]. Due to the limitation of the previous studies, the detailed diagnostic power of urine Fn in the detection of BCa still needs to be investigated. For this reason, we conducted the meta-analysis to pool all the eligible studies to fully evaluate the real and detailed diagnostic performance of urine Fn for bladder cancer.

In the present meta-analysis, we included eight individual studies, containing 744 bladder cancer patients. Our meta-analysis showed that urine Fn had a relatively high diagnostic value, with an AUC of 0.83, a Q* index of 0.798 and a rather restricted confidence region and prediction region. The pooled sensitivity and specificity of urine Fn were 0.80 and 0.79, respectively, which were nearly the same. Besides, the overall DOR was 15.18. Although the PLR and NLR of this biomarker seemed not to be so outstanding, based on the above-mentioned good results, we still concluded that urine Fn could be a powerful biomarker for detecting BCa. Besides, a certain degree of heterogeneity was observed in this meta-analysis, especially in Spe and DOR. Accordingly, the meta-regression showed that measurement units, pathological types, assay methods, and the proportion of NMIBC cases were the main source of heterogeneity.

In consideration of the influences of confounding factors on diagnostic accuracy, subgroup analysis was carried out on the basis of some common covariates and the results were complicated. First, some investigators tried to use urine creatinine or some other calibrators to adjust the urine Fn level in order to correct the differences caused by urine concentrations [19, 21–23]. Thus, in some researches, the urine Fn/Cr ratio was widely used to replace the urine Fn concentration (μg/L). The subgroup analysis, however, did not show any improvement of the diagnostic accuracy in the “non-μg/L” subgroup. Instead, the group using the original Fn concentration as the measurement unit had a much better diagnostic performance than the other group, with an AUC of 0.88 (0.80–0.94) versus 0.80 (0.76–0.83). We all know that the urine creatinine to a great extent depends on the renal function of different patients, so choosing it to adjust the urine Fn concentration might lead to more complicated and vague outcomes rather than more precise results. Therefore, the original urine Fn concentration was recommended for the future researches for the sake of more reliable results. Moreover, the studies based on both BTCC and SCC had a larger AUC (0.89) than that of the studies based on BTCC alone (0.86), indicating that urine Fn also had potential diagnostic value for SCC and might be popularized in the high incidence area of bladder SCC, such as Egypt. In addition, to our knowledge, the urine Fn level might be significantly higher in MIBC patients than that in NMIBC patients [19, 20]. Interestingly, the pooled analysis of the “NMIBC > 50%” group exhibited better diagnostic performances. This result indicated that the urine Fn could be used as a biomarker for both NMIBC and MIBC cases. More researches are needed so as to compare the diagnostic performance of urine Fn in NMIBC patients.

Furthermore, we also collected data of a combined method (urine Fn combined with urine cytology). The pooled sensitivity, specificity, DOR, diagnostic score, PLR, NLR, and AUC was 0.86 (95%CI = 0.82–0.90), 0.77 (95%CI = 0.70–0.84), 21.20 (95%CI = 14.30–31.44), 3.05 (95%CI = 2.66–3.45), 3.82 (95%CI = 2.87–5.08), 0.18 (95%CI = 0.13–0.24), and 0.89 (95%CI = 0.86–0.92), respectively, and the *Z* tests showed that the AUC and the sensitivity of the combined methods were significantly higher. These results suggested that the performance of urine Fn to detect bladder cancer could be significantly improved in combination with urine cytology. Regarding the widely reported “Achilles’ heel” of urine cytology—the rather low sensitivity, this combined method (Fn+Cyto) appeared to enhance the sensitivity for both Fn alone and cytology alone. In addition, the application of the two tests in combination would become a potential alternative choice to partly displace the invasive cystoscopic evaluations.

Besides, the limitations of this meta-analysis were as follows. First, a not small heterogeneity was observed in this meta-analysis, especially in the Spe and DOR. Although we tried to explore the sources of the heterogeneity via performing Spearman rank correlation test, univariable meta-regression and subgroup analysis, the heterogeneity of DOR was still obvious. We noticed that the *I*^2^ of the DOR were slightly decreased in “BTCC & SCC” group, so we think the heterogeneity of DOR might come from the different pathological types. Second, the included bladder cancer cases were less than 1000 patients and the limited sample size could influence the analytical power. Third, due to the various units of measurement and the diverse values in different studies, we could not give a pooled cut-off value of urine Fn in detecting bladder cancer. The specific cut-off value for clinical use can vary from country to country and from hospital to hospital, which should be confirmed by clinical practice based on a large population. Finally, the diagnostic biomarker of urine Fn has not been widely applied in clinical practice. Future studies should focus on how to improve the diagnostic power of urine Fn and how to test urine Fn more efficiently and easily. More and more large, multi-center and prospective studies are needed in order to validate the diagnostic power of urine Fn.

## Conclusions

In conclusion, by evaluating the pooled sensitivity, specificity, PLR, NLR, DOR, and AUC from 11 studies, the present meta-analysis revealed that measurement of urine fibronectin appears to be relatively useful for the detection of bladder cancer. And this biomarker could also be potentially applied to SCC and NMIBC. Meta-analysis of the combined methods indicates the combination of urine Fn and urine cytology significantly enhances the sensitivity and diagnostic performance and has the ability to become the alternative diagnostic test in clinical practice.

## Additional files

Additional file 1: **Figure S1.** QUADAS-2 assessments for the risk of bias of the included studies. (A) Risk of bias and applicability concerns graph. (B) Risk of bias and applicability concerns summary. (TIFF 363 kb)

Additional file 2: **Figure S2.** Univariable meta-regression plot for (A) sensitivity and (B) specificity of urine Fn. (TIFF 330 kb)

## Abbreviations

AUC: Area under curve
BCa: Bladder cancer
BSA: Bovine serum albumin
BTA: Bladder tumor antigen
BTCC: Bladder transitional cell carcinoma
CBM: China Biology Medicine disc
CI: Confidence interval
Cr: Creatinine
Cyto: Urine cytology
DOR: Diagnostic odds ratio
ELISA: Enzyme linked immunosorbent assay
FDP: Fibrinogen degradation product
FN: False negative
Fn: Fibronectin
FP: False positive
HSROC: Hierarchical summary receiver operator curve
MIBC: Muscle-invasive bladder cancer
NLR: Negative likelihood ratio
NMIBC: Non-muscle-invasive bladder cancer
NMP 22: Nuclear matrix protein 22
NR: Not reported
PLR: Positive likelihood ratio
QUADAS-2: Quality assessment of diagnostic accuracy studies-2
QUOROM: Quality of Reporting of Meta-analysis
ROC: Receiver operating characteristic
SCC: Squamous cell carcinoma
Sen: Sensitivity
Spe: Specificity
SROC: Summary receiver operating characteristic
TN: True negative
TP: True positive
TURBT: Transurethral resection of bladder tumor
