# Supplementary figures and images for: Effectiveness of urine fibronectin as a non-invasive diagnostic biomarker in bladder cancer patients: a systematic review and meta-analysis
Source: World J Surg Oncol. 2018 Mar 21;16:61. doi: 10.1186/s12957-018-1358-x (PMC5863379; doi:10.1186/s12957-018-1358-x)

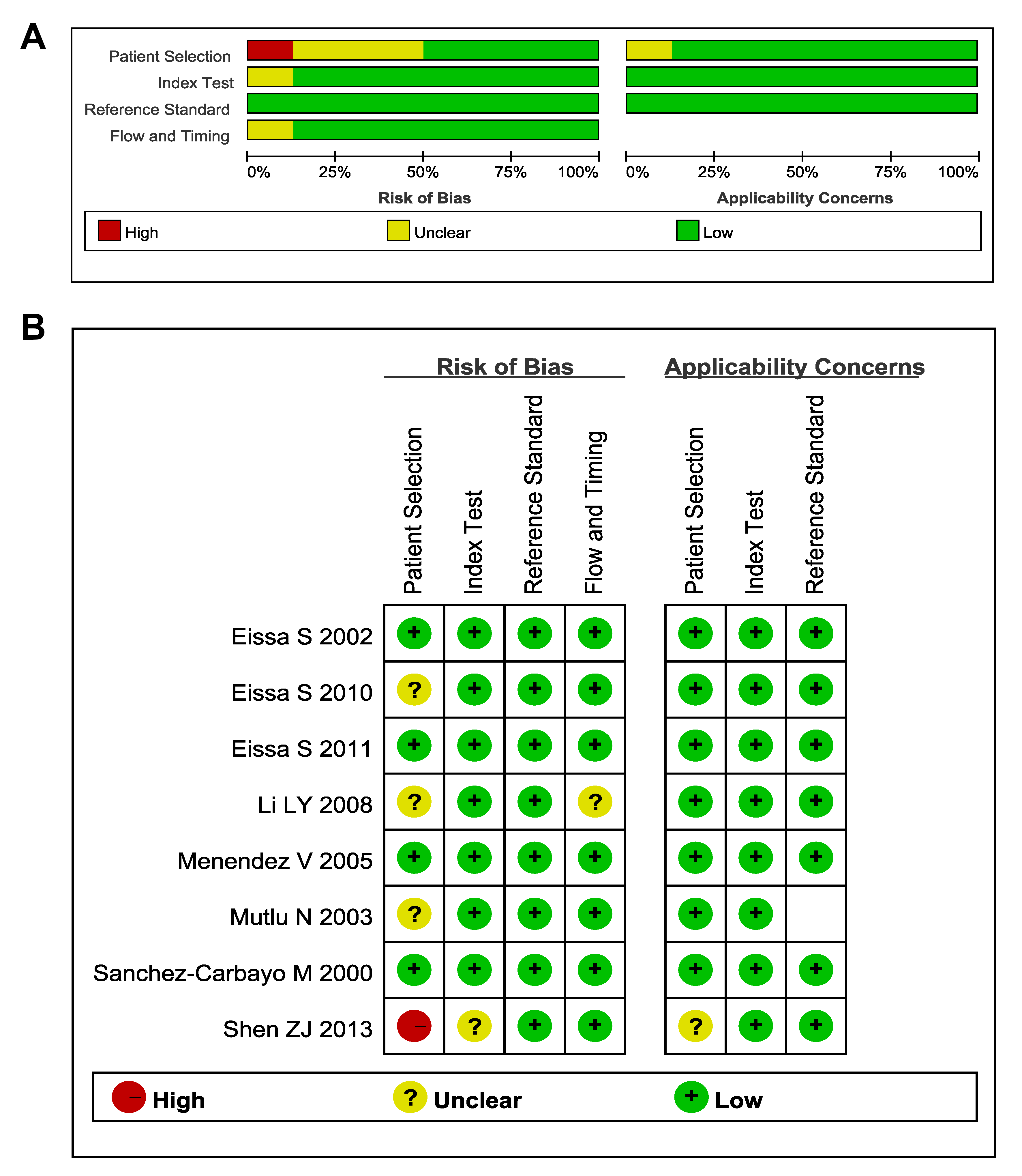

Supplement: Supplementary file 1 — Figure S1. QUADAS-2 assessments for the risk of bias of the included studies. (A) Risk of bias and applicability concerns graph. (B) Risk of bias and applicability concerns summary. (TIFF 363 kb) [file 12957_2018_1358_MOESM1_ESM.tif]

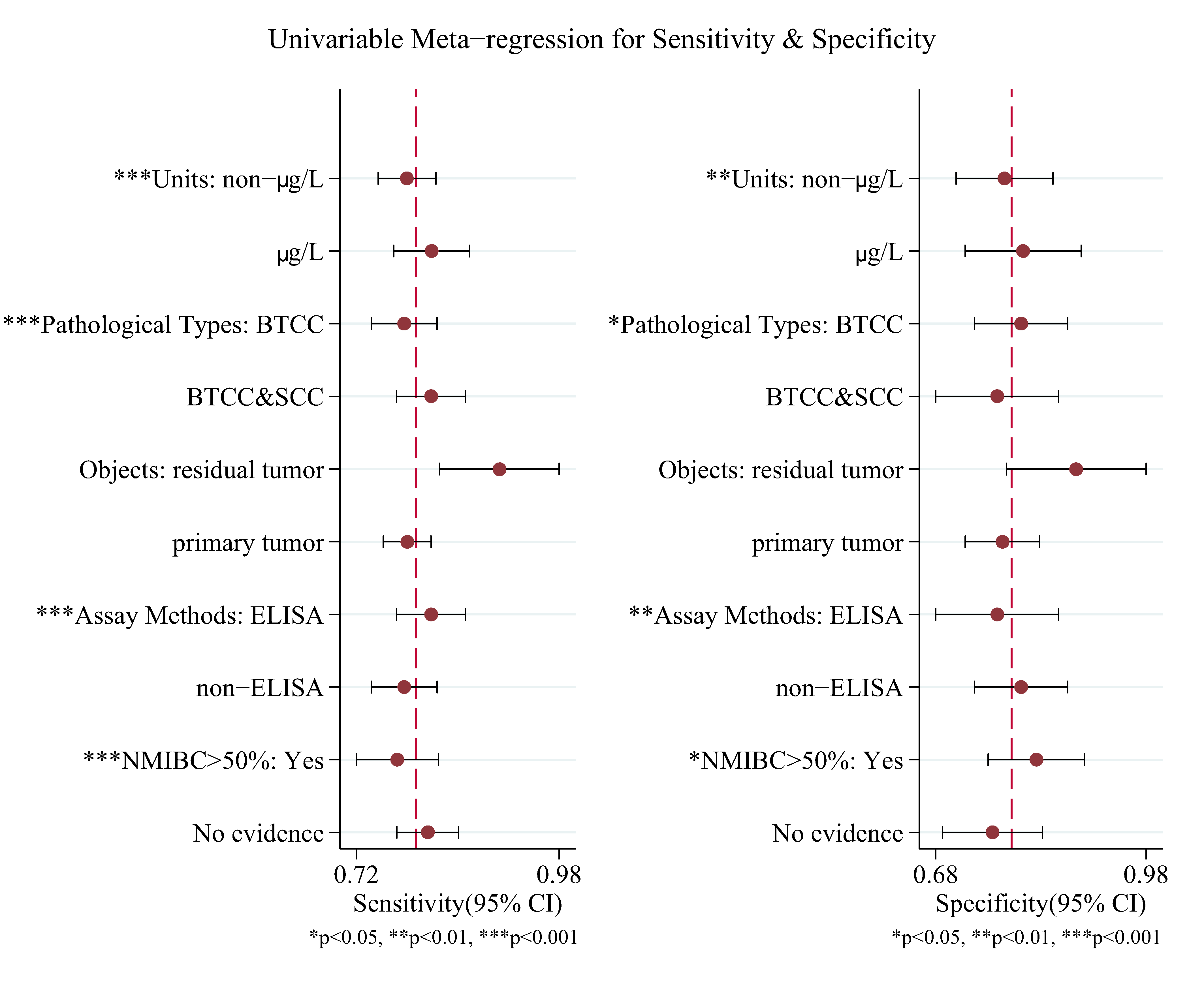

Supplement: Supplementary file 2 — Figure S2. Univariable meta-regression plot for (A) sensitivity and (B) specificity of urine Fn. (TIFF 330 kb) [file 12957_2018_1358_MOESM2_ESM.tif]
